# Genomic analysis of a novel pathogenic variant in the gene LMNA associated with cardiac laminopathies found in Ecuadorian siblings: A case report

**DOI:** 10.3389/fcvm.2023.1141083

**Published:** 2023-03-21

**Authors:** Patricia Guevara-Ramírez, Santiago Cadena-Ullauri, Rita Ibarra-Castillo, José Luis Laso-Bayas, Elius Paz-Cruz, Rafael Tamayo-Trujillo, Viviana A. Ruiz-Pozo, Nieves Doménech, Adriana Alexandra Ibarra-Rodríguez, Ana Karina Zambrano

**Affiliations:** ^1^ Centro de Investigación Genética y Genómica, Facultad de Ciencias de la Salud Eugenio Espejo, Universidad UTE, Quito, Ecuador; ^2^ Department of Hemodynamics, Clinical Cardiac Electrophysiologist, Quito-Ecuador, Ecuador; ^3^ Instituto de Investigación Biomédica de A Coruña (INIBIC)-CIBERCV, Complexo Hospitalario Universitario de A Coruña (CHUAC), Sergas, Universidad da Coruña (UDC), La Coruña-Spain, Spain; ^4^ Grupo de Investigación Identificación Genética-IdentiGEN, FCEN, Universidad de Antioquia, Medellin-Colombia, Colombia

**Keywords:** cardiovascular disease, genome, precision medicine, NGS, LMNA

## Abstract

**Introduction:**

Cardiac laminopathies are caused by mutations in the LMNA gene and include a wide range of clinical manifestations involving electrical and mechanical changes in cardiomyocytes. In Ecuador, cardiovascular diseases were the primary cause of death in 2019, accounting for 26.5% of total deaths. Cardiac laminopathy-associated mutations involve genes coding for structural proteins with functions related to heart development and physiology.

**Family description:**

Two Ecuadorian siblings, self-identified as mestizos, were diagnosed with cardiac laminopathies and suffered embolic strokes. Moreover, by performing Next-Generation Sequencing, a pathogenic variant (NM_170707.3:c.1526del) was found in the gene LMNA.

**Discussion and conclusion:**

Currently, genetic tests are an essential step for disease genetic counseling, including cardiovascular disease diagnosis. Identification of a genetic cause that may explain the risk of cardiac laminopathies in a family can help the post-test counseling and recommendations from the cardiologist. In the present report, a pathogenic variant ((NM_170707.3:c.1526del) has been identified in two Ecuadorian siblings with cardiac laminopathies. The LMNA gene codes for A-type laminar proteins that are associated with gene transcription regulation. Mutations in the LMNA gene cause laminopathies, disorders with diverse phenotypic manifestations. Moreover, understanding the molecular biology of the disease-causing mutations is essential in deciding the correct type of treatment.

## Introduction

The lamin A/C gene (LMNA), located on chromosome 1q22, encodes two proteins on the inner face of the nuclear membrane: lamins A and C. Lamins A/C are expressed in almost all cell types, including cardiomyocytes (1, 2). Thus, several mutations in the LMNA gene have been associated with cardiac phenotypes characterized by different clinical manifestations, including structural abnormalities and electrical and mechanical instability of cardiomyocytes (3). As a result, dilated cardiomyopathy (DCM) is the most common phenotype, as well as other clinical manifestations such as atrioventricular conduction abnormalities, including atrial and ventricular tachyarrhythmias. In addition, severe heart failure or sudden cardiac death may occur in several cases (2).

The epidemiology of cardiac laminopathies is still unknown in Ecuador; however, cardiovascular diseases were the first cause of death in 2019, accounting for 26.5% of total deaths in the country (4).

**Figure 1:**
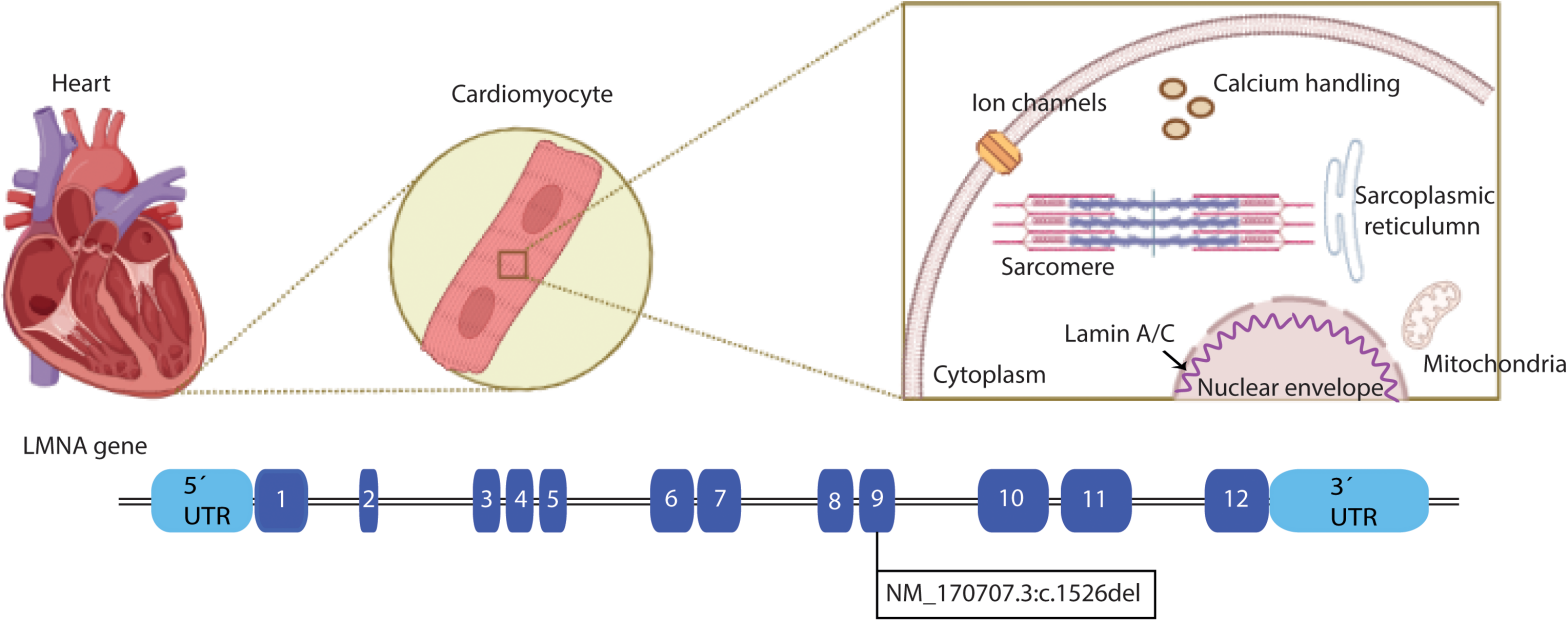
Schematic structure of LMNA. In the upper panel, a heart is depicted, zooming in on the cardiomyocytes and the cellular location of the Lamin A/C proteins. The LMNA gene is represented in the lower panel, and each filled box indicates the exons of the gene. The LMNA pathogenic variant NM_170707.3:c.1526del is located at exon 9 (Created with BioRender.com).

Cardiac laminopathies are associated with mutations in the LMNA gene, including nonsense and splice mutations, insertions, and deletions. In the present case report, a frameshift mutation caused by a deletion was found in the LMNA gene (Figure 1), the most mutated gene in cardiac laminopathies. The LMNA is composed of 12 exons. Lamin A and C are synthesized by alternative splicing of exon 10. Lamins are V-type intermediate filaments located exclusively in the nucleus of cells (5). Lamin A is translated as a precursor, prelamin A, and requires extensive C-terminal processing to mature, whereas lamin C is translated as a mature protein. The lamina joined to nuclear envelope spanning proteins *via* the linker of nucleoskeleton and cytoskeleton complex is critical for whole-cell mechanics and mechano-transduction to the nucleus (6). Other mutations associated with cardiac laminopathies involve genes coding for structural proteins of the cardiac cytoskeleton, sarcomere components, intercellular junctions, ion channels, mitochondrial proteins, nuclear lamina proteins, intermediate filaments, and the dystrophin-associated glycoprotein complex (3, 7).

This case report presents two Ecuadorian siblings diagnosed with cardiac laminopathies who had embolic strokes. Clinical, genomic, protein-protein interaction (PPI) network, and gene ontology enrichment (GO) analyses were performed to provide evidence of the cardiac pathology, associated phenotypes, and molecular aspects. The information collected by Next-Generation Sequencing (NGS) could be a key aspect in cardiovascular disease diagnosis and treatment.

## Family description

The subjects presented in this case report are two siblings, self-identified as mestizos, from Sigchos in Cotopaxi, Ecuador. Subject A is a 58-year-old woman who, seven years ago, had an embolic stroke that left her with a 46% physical disability and with walking and speaking difficulties. Four years ago, she underwent a cardiac catheterization that showed myocardial fibrosis compatible with cardiac laminopathies. In the last year, she underwent pacemaker and defibrillator surgeries due to congestive heart failure, which increased the risk of sudden death. Subject A takes warfarin to prevent blood clots.

**Figure 2:**
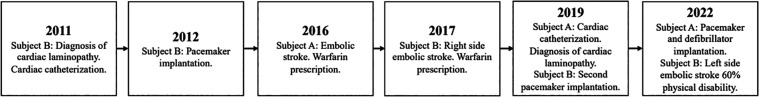
Timeline of subjects’ relevant episodes. The disease-associated episodes related to the subjects.

Subject B is a 51-year-old man diagnosed with cardiac laminopathies 12 ago after cardiac catheterization. Six years later, the subject suffered a right-sided embolic stroke, and five years later, he suffered a left-sided embolic stroke, leaving him with a 60% physical disability, including speaking and walking difficulties, similar to his sister. Moreover, he has suffered from severe arrhythmic attacks, which led to a pacemaker surgery to control the heartbeat and a second pacemaker surgery to replace the device's batteries. Subject B takes warfarin for blood clot prevention. A timeline with the relevant points of care episodes is depicted in Figure 2.

**Figure 3:**
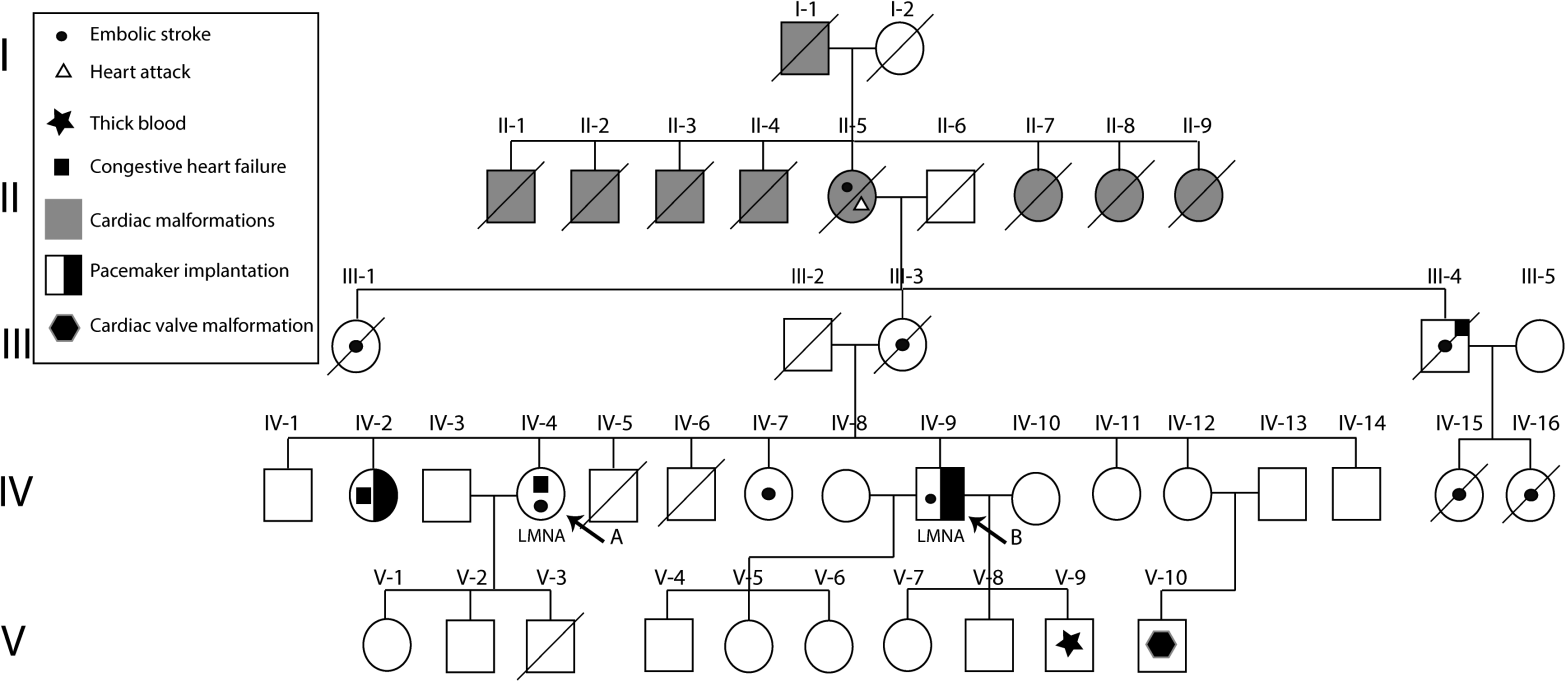
Family pedigree of reported subjects with hereditary cardiac laminopathies. Subjects (arrow) carry a pathogenic variant, NM_170707.3:c.1526del, in the LMNA gene. Furthermore, multiple family members presented thrombosis, heart attacks, thick blood, atrioventricular blocks, and cardiac malformations. Likewise, two of the family members underwent pacemaker implantation.

Furthermore, their family tree is depicted in Figure 3. Subjects II-1 to II-9 suffered from cardiac malformations, heart attacks, and thrombosis that due to the health care system conditions at the time, could not be distinctly diagnosed. Subjects II-5, III-1, III-3, III-4, IV-15, and IV-16 died from embolic strokes when they were under 60. Subject IV-2 has undergone pacemaker surgery because she showed an atrioventricular block, thrombosis, and arrhythmias. Subject V-10 presents a cardiac valve malformation; however, further testing is required.

## Results

**Figure 4:**
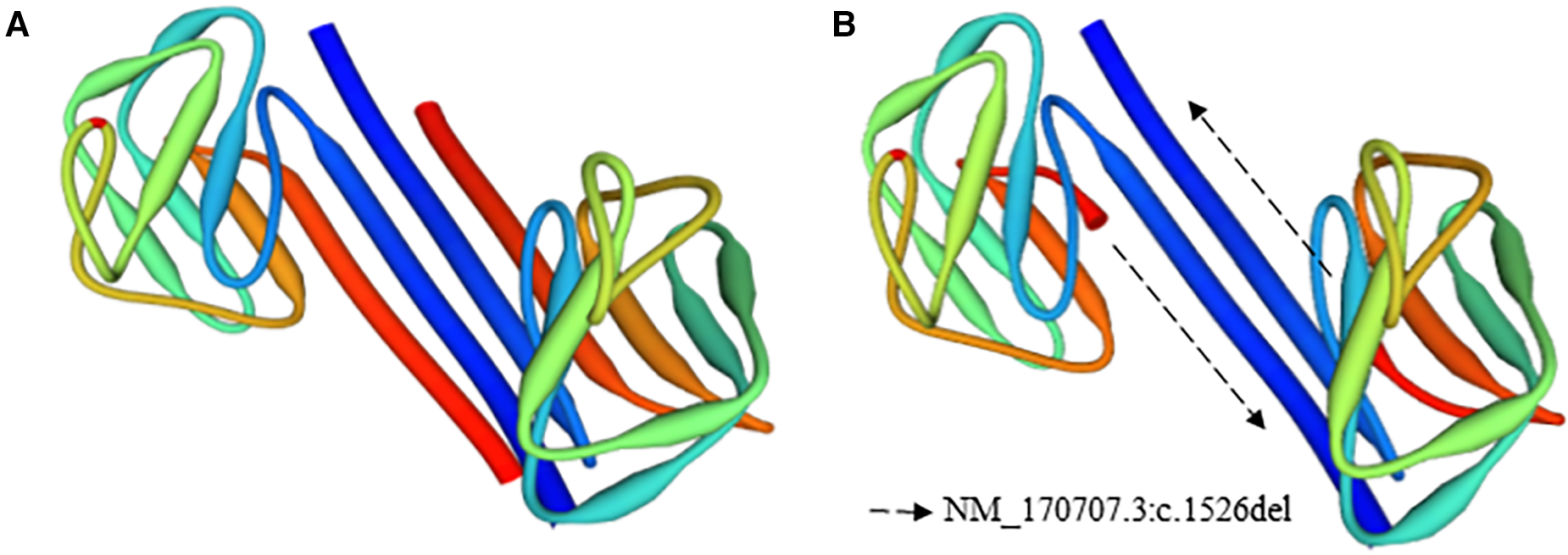
3D structure of the native and mutant LMNA protein models. (**A**) Native, (**B**) mutant model.

NGS was performed using the TruSight Cardio Kit from Illumina®; this panel includes 174 genes associated with 17 inherited cardiac disorders (Supplementary Table S1). In subjects A and B, 98% of the targets had 50 times or more coverage. Both patients exhibited variants, which were categorized as benign, likely benign, variant of unknown significance (VUS), and pathogenic, according to BSKN (BaseSpace Knowledge Network, Illumina ®, San Diego, CA) (Supplementary Table S2). For the analyses, the VUS and pathogenic variants were taken into consideration. Variants of unknown significance were detected in the genes KCNH2, PRDM16, and HCN4. Moreover, a pathogenic variant (NM_170707.3:c.1526del) was found in the gene LMNA of subjects A and B. Furthermore, NGS was performed on Subject V-2, and no pathogenic mutations were identified. The 3D structures of the native and the mutant LMNA protein were drawn using the PyMol molecular graphics system to provide a clear image of the deletion in Figure 4.

A STRING protein-protein interaction network analysis was performed, as presented in Supplementary Figure S1. The chosen proteins have been reported to have a role in cardiac laminopathies. Furthermore, the Biological Process (Gene Ontology) on which the proteins coded by LMNA had a role and an association with heart development and physiology were selected. The network type was the Full STRING, the thickness of the edges indicates the confidence, and the minimum required interaction score was high confidence (0.700). The chosen biological processes were regulation of muscle system process, muscle organ development, muscle structure development, heart development, cardiac muscle cell development, mitotic nuclear envelope reassembly, cardiac muscle tissue development, striated muscle cell development, cardiac muscle cell differentiation, and striated muscle cell differentiation. Moreover, a functional analysis was carried out to identify different molecular function processes, KEGG pathways, and phenotypes with which LMNA has been associated, as presented in Supplementary Figure S2. Each term has been marked with an ID. The significance threshold was calculated using the g:SCS method, where the lower the *p*_adj_ value, the more significant the association. The terms with the highest significance associated with LMNA were dilated cardiomyopathy, sudden cardiac death, myopathy, ventricular arrhythmia, abnormal left ventricular function, abnormal cardiovascular system physiology, arrhythmia, heart block, abnormality of cardiovascular system morphology, and cardiac conduction abnormality.

## Discussion

Currently, genetic tests are an essential step for disease genetic counseling (8, 9). For example, different studies suggest that genetic testing can identify a disease-causing variant in approximately 15%–25% of patients with sporadic dilated cardiomyopathy (DCM) and about 20%–40% of patients with familial DCM (5, 10–12). However, NGS has limitations; for instance, the need for bioinformatic tools for data analysis or the limited information regarding variants of uncertain significance. Similarly, although NGS costs have decreased over the last few years, they are still unreachable to most of the population. The problem is exacerbated in regions with a high percentage of inequality, such as Latin America, where access to healthcare systems is limited even today (13).

Identification of a genetic cause that may explain the risk of cardiac laminopathies in a family can help the post-test counseling and recommendations from the cardiologist. Wilsbacher suggests four benefits of genetic testing: (1) it can provide a diagnosis; (2) it can identify family members at risk for developing cardiomyopathy; (3) genetic test results can be used for prenatal genetic counseling and preimplantation genetic diagnosis; and (4) medical treatment can change. For example, patients with LMNA mutations have a high burden of ventricular tachycardia, so exercise must be restricted and an implantable cardioverter defibrillator (ICD) is recommended (14).

The LMNA gene codes for A-type laminar proteins, mainly Lamin A and C (3, 15); these are key factors in gene transcription regulation by modulating DNA replication, signal transduction, and chromatin organization (7). Mutations in the LMNA gene cause laminopathies (3), and the heart is one of the most affected organs when an LMNA mutation is present. Fatkin et al. (1999) were among the first to describe the role of LMNA mutations in cardiovascular disorders; since then, several other mutations in the LMNA gene have been associated with laminopathies (3, 16–20). Moreover, mutations in the LMNA gene result in severe nuclear abnormalities and a higher risk of thromboembolic complications (18, 19). Patients carrying LMNA mutations present more aggressive laminopathies and have higher rates of life-threatening arrhythmias and end-stage heart failure (21–23).

In the present case report, a pathogenic variant (NM_170707.3:c.1526del) in the LMNA gene was successfully identified; this is the first report of the pathogenic variant in the LMNA gene in Ecuadorian subjects with cardiac laminopathies. Similarly, Pasotti M. et al. (2008) found the same deletion in a patient with atrioventricular block, dilated cardiomyopathy, and Emery-Dreifuss muscular dystrophy type 2. The subjects described in this case report did not have any Emery-Dreifuss muscular dystrophy-related symptoms; however, their cardiac phenotype was similar to the phenotype of the patients diagnosed with dilated cardiomyopathy.

The 3D structure of the native and mutant protein, as presented in Figure 4, reflects the impact of the nonsense mutation on the final protein conformation. Additionally, the reduction of the LMNA expression level could be increased by the nonsense-mediated mRNA decay control mechanisms (24–26). During this process, the incomplete aberrant mRNA was eliminated to maintain the integrity of gene expression and to avoid the accumulation of these mRNAs (26). Furthermore, the STRING network, seen in Supplementary Figure S1, represents genes associated with cardiac laminopathies based on the biological process (Gene Ontology) (27). The colors represent the LMNA-related processes. It is important to highlight the relevance of the gene, as depicted in the figure, in heart development and physiology.

Moreover, Supplementary Figure S2 indicates the molecular function, KEGG pathways, and the human phenotype ontology of the same genes. The processes where LMNA was involved are represented in the figure, emphasizing cardiac disorders such as cardiac arrest, sudden death, arrhythmia, heart block, dilated cardiomyopathy, and tachycardia, among others.

A limitation of the present study is that due to budget constraints, we were unable to perform NGS in all the family members; however, we present solid results, including bioinformatic analyses, of why the reported variant could be associated with an affected cardiac phenotype.

## Conclusion

Mutations in the LMNA gene have been broadly described and associated with heart diseases. In the present case report, a pathogenic variant has been identified in two Ecuadorian siblings with a family history of embolic strokes, heart attacks, heart failure, and cardiac malformations. Moreover, understanding the molecular biology of diseases could guide physicians to decide the proper type of treatment.

## Data Availability

The datasets presented in this article are not readily available because of ethical and privacy restrictions. Requests to access the datasets should be directed to the corresponding author

## References

[1] Gerbino A Procino G Svelto M Carmosino M. Role of lamin A/C gene mutations in the signaling defects leading to cardiomyopathies. Front Physiol. (2018) 9:1–7. 10.3389/fphys.2018.01356 30319452 PMC6167438

[2] Peretto G Sala S Benedetti S Di Resta C Gigli L Ferrari M Updated clinical overview on cardiac laminopathies: an electrical and mechanical disease. Nucleus. (2018) 9:380–91. 10.1080/19491034.2018.1489195 29929425 PMC7000139

[3] Crasto S My I Di Pasquale E. The broad Spectrum of LMNA cardiac diseases: from molecular mechanisms to clinical phenotype. Front Physiol. (2020) 11:1–11. 10.3389/fphys.2020.00761 32719615 PMC7349320

[4] Organización Pan American de la Salud. Iniciativa Regional De Datos Básicos en Salud. Generador de Tablas. Glosario en línea (2022).

[5] Rosenbaum AN Agre KE. Genetics of dilated cardiomyopathy: practical implications for heart failure management. Nat Rev Cardiol. (2020) 17:286–97. 10.1038/s41569-019-0284-0 31605094

[6] Brayson D Shanahan CM. Current insights into LMNA cardiomyopathies: existing models and missing LINCs. Nucleus. (2017) 8:17–33. 10.1080/19491034.2016.1260798 28125396 PMC5287098

[7] McNally EM Mestroni L. DCM: genetic determinants and mechanisms. Circ Res. (2017) 121:731–48. 10.1161/CIRCRESAHA.116.309396 28912180 PMC5626020

[8] Kalayinia S Goodarzynejad H Maleki M Mahdieh N. Next generation sequencing applications for cardiovascular disease. Ann Med. (2018) 50:91–109. 10.1080/07853890.2017.1392595 29027470

[9] Cadena-Ullauri S Guevara-Ramirez P Ruiz-Pozo V Tamayo-Trujillo R Paz-Cruz E Insuasty TS Case report: genomic screening for inherited cardiac conditions in Ecuadorian mestizo relatives: improving familial diagnose. Front Cardiovasc Med. (2022) 9:1037370. 10.3389/fcvm.2022.1037370 36426223 PMC9678921

[10] Hershberger RE Morales A Siegfried J. Clinical and genetic issues in dilated cardiomyopathy: a review for genetics professionals. Genet Med. (2011) 12:655–67. 10.1097/GIM.0b013e3181f2481f PMC3118426 20864896

[11] Malakootian M Moghaddam MB Kalayinia S Farrashi M Maleki M Sadeghipour P Dilated cardiomyopathy caused by a pathogenic nucleotide variant in RBM20 in an Iranian family. BMC Med Genomics. (2022) 15:1–9. 10.1186/s12920-022-01262-4 35527250 PMC9079971

[12] Sweet M Taylor MRG Mestroni L Genetics AM Genetics M Program G. Diagnosis, prevalence, and screening of familial dilated cardiomyopathy. Expert Opin Orphan Drugs. (2016) 3:869–76. 10.1517/21678707.2015.1057498 PMC4988677 27547593

[13] Ruano AL Rodríguez D Rossi PG Maceira D. Understanding inequities in health and health systems in Latin America and the Caribbean: a thematic series. Int J Equity Health. (2021) 20:1–4. 10.1186/s12939-021-01426-1 33823879 PMC8023548

[14] Wilsbacher LD. Clinical implications of the genetic architecture of dilated cardiomyopathy. Curr Cardiol Rep. (2020) 22:170. 10.1007/s11886-020-01423-w 33040239 PMC7547954

[15] Dittmer T Misteli T. The lamin protein family. Genome Biol. (2011) 12:222. 10.1186/gb-2011-12-5-222 21639948 PMC3219962

[16] Jakobs PM Hanson EL Crispell KA Toy W Keegan H Schilling K Novel lamin A/C mutations in two families with dilated cardiomyopathy and conduction system disease. J Card Fail. (2001) 7:249–56. 10.1054/jcaf.2001.26339 11561226

[17] Hershberger RE Siegfried JD. Clinical and genetic issues in familial dilated cardiomyopathy. J Am Coll Cardiol. (2011) 57:1641–9. 10.1016/j.jacc.2011.01.015.Update 21492761 PMC3088091

[18] Saga A Karibe A Otomo J Iwabuchi K Takahashi T Kanno H Lamin A/C gene mutations in familial cardiomyopathy with advanced atrioventricular block and arrhythmia. Tohoku J Exp Med. (2009) 218:309–16. 10.1620/tjem.218.309 19638735

[19] van Tintelen JP Hofstra RMW Katerberg H Rossenbacker T Wiesfeld ACP du Marchie Sarvaas GJ High yield of LMNA mutations in patients with dilated cardiomyopathy and/or conduction disease referred to cardiogenetics outpatient clinics. Am Heart J. (2007) 154:1130–9. 10.1016/j.ahj.2007.07.038 18035086

[20] van Tintelen JP Tio RA Kerstjens-Frederikse WS van Berlo JH Boven LG Suurmeijer AJH Severe myocardial fibrosis caused by a deletion of the 5′ end of the lamin A/C gene. J Am Coll Cardiol. (2007) 49:2430–9. 10.1016/j.jacc.2007.02.063 17599607

[21] Van Rijsingen IAW Bakker A Azim D Hermans-Van Ast JF Van Der Kooi AJ Van Tintelen JP Lamin A/C mutation is independently associated with an increased risk of arterial and venous thromboembolic complications. Int J Cardiol. (2013) 168:472–7. 10.1016/j.ijcard.2012.09.118 23073275

[22] Rhoades JH Prosser BL Musunuru K. Pathogenic LMNA variants disrupt cardiac lamina-chromatin interactions and de-repress alternative fate genes ll article pathogenic LMNA variants disrupt cardiac lamina-chromatin interactions and de-repress alternative fate genes. Stem Cell. (2021) 28:938–54.e9. 10.1016/j.stem.2020.12.016 PMC8106635 33529599

[23] Ferradini V Cosma J Romeo F De MC Murdocca M Spitalieri P Clinical features of LMNA-related cardiomyopathy in 18 patients and characterization of two novel variants. J Clin Med. (2021) 10:1–14. 10.3390/jcm10215075 PMC8584896 34768595

[24] Baker KE Parker R. Nonsense-mediated mRNA decay : terminating erroneous gene expression. Curr Opin Biol. (2004) 16:293–9. 10.1016/j.ceb.2004.03.003 15145354

[25] Brogna S Wen J. Nonsense-mediated mRNA decay (NMD) mechanisms. Nat Struct Mol Biol. (2009) 16:107–13. 10.1038/nsmb.1550 19190664

[26] Kurosaki T Popp MW Maquat LE. Quality and quantity control of gene expression by nonsense-mediated mRNA decay. Nat Rev Mol Cell Biol. (2019) 20:406–20. 10.1038/s41580-019-0126-2 30992545 PMC6855384

[27] Li M Xia S Xu L Tan H Yang J Wu Z Genetic analysis using targeted next-generation sequencing of sporadic Chinese patients with idiopathic dilated cardiomyopathy. J Transl Med. (2021) 19:1–8. 10.1186/s12967-021-02832-3 33941202 PMC8091742

