# Supplementary material for: Genomic analysis of a novel pathogenic variant in the gene LMNA associated with cardiac laminopathies found in Ecuadorian siblings: A case report
Source: Front Cardiovasc Med. 2023 Mar 21;10:1141083. doi: 10.3389/fcvm.2023.1141083 (PMC10070725; doi:10.3389/fcvm.2023.1141083)
Supplement: Supplementary file 1 [file Table1.docx]

| **N°** | **Inherited cardiac disorders** | **Genes** |
| --- | --- | --- |
| 1 | Aortic Valve Disease | *ABCC9, ABCG5, ABCG8, ACTA1, ACTA2, ACTC1, ACTN2, AKAP9, ALMS1, ANK2, ANKRD1, APOA4, APOA5, APOB, APOC2, APOE, BAG3, BRAF, CACNA1C, CACNA2D1, CACNB2, CALM1, CALR3, CASQ2, CAV3, CBL, CBS, CETP, COL3A1, COL5A1, COL5A2, COX15, CREB3L3, CRELD1, CRYAB, CSRP3, CTF1, DES, DMD, DNAJC19, DOLK, DPP6, DSC2, DSG2, DSP, DTNA, EFEMP2, ELN, EMD, EYA4, FBN1, FBN2, FHL1, FHL2, FKRP, FKTN, FXN, GAA, GATAD1, GCKR, GJA5, GLA, GPD1L, GPIHBP1, HADHA, HCN4, HFE, HRAS, HSPB8, ILK, JAG1, JPH2, JUP, KCNA5, KCND3, KCNE1, KCNE2, KCNE3, KCNH2, KCNJ2, KCNJ5, KCNJ8, KCNQ1, KLF10, KRAS, LAMA2, LAMA4, LAMP2, LDB3, LDLR, LDLRAP1, LMF1, LMNA, LPL, LTBP2, MAP2K1, MAP2K2, MIB1, MURC, MYBPC3, MYH11, MYH6, MYH7, MYL2, MYL3, MYLK, MYLK2, MYO6, MYOZ2, MYPN, NEXN, NKX2-5, NODAL, NOTCH1, NPPA, NRAS, PCSK9, PDLIM3, PKP2, PLN, PRDM16, PRKAG2, PRKAR1A, PTPN11, RAF1, RANGRF, RBM20, RYR1, RYR2, SALL4, SCN1B, SCN2B, SCN3B, SCN4B, SCN5A, SCO2, SDHA, SEPN1, SGCB, SGCD, SGCG, SHOC2, SLC25A4, SLC2A10, SMAD3, SMAD4, SNTA1, SOS1, SREBF2, TAZ, TBX20, TBX3, TBX5, TCAP, TGFB2, TGFB3, TGFBR1, TGFBR2, TMEM43, TMPO, TNNC1, TNNI3, TNNT2, TPM1, TRDN, TRIM63, TRPM4, TTN, TTR, TXNRD2, VCL, ZBTB17, ZHX3, ZIC3* |
| 2 | Marfan Syndrome |  |
| 3 | Loeys-Dietz Syndrome |  |
| 4 | Short QT Syndrome |  |
| 5 | Catecholaminergic Polymorphic Ventricular Tachycardia |  |
| 6 | Familial Hypercholesterolemia |  |
| 7 | Restrictive Cardiomyopathy |  |
| 8 | Non-Compaction Cardiomyopathy |  |
| 9 | Noonan Syndrome |  |
| 10 | Arrhythmogenic Right Ventricular Cardiomyopathy (ARVC) |  |
| 11 | Brugada Syndrome |  |
| 12 | Structural Heart Disease |  |
| 13 | Long QT Syndrome |  |
| 14 | Familial Aortic Aneurysm |  |
| 15 | Familial Atrial Fibrillation |  |
| 16 | Hypertrophic Cardiomyopathy |  |
| 17 | Dilated Cardiomyopathy |  |

**Supplementary Table 1:** Inherited cardiac conditions and genes associated included in the TruSight Cardio Sequencing Panel.
