# Supplementary material for: Genomic analysis of a novel pathogenic variant in the gene LMNA associated with cardiac laminopathies found in Ecuadorian siblings: A case report
Source: Front Cardiovasc Med. 2023 Mar 21;10:1141083. doi: 10.3389/fcvm.2023.1141083 (PMC10070725; doi:10.3389/fcvm.2023.1141083)
Supplement: Supplementary file 2 [file Table2.docx]

**Supplementary Table 2:** Shared variants between subjects.

| **Gene** | **Description** | **Cygozity** | **Association** | **Subject** |
| --- | --- | --- | --- | --- |
| *LMNA* | Frameshift Indels NM_170707.3 c.1526del p.(Pro509Leu*fs*Ter39)  Exón: 9/12 | Heterozygous | Pathogenic | A and B |
| *KCNH2* | Missense NM_000238.3 c.1342G>A p.(Ala448Thr) Exon: 6/15 | Heterozygous | Variant of uncertain significance (VUS) * | A and B |
| *PRDM16* | Missense NM_022114.3 c.787G>A p.(Gly263Ser) Exon: 6/17 | Heterozygous | Variante de significado incierto (VUS) * | A, B, Subject V-2 |
| *HCN4* | Missense NM_005477.2 c.152G>A p.(Arg51Gln) Exon: 1/8 | Heterozygous | Variante de significado incierto (VUS) * | A, B, Subject V-2 |
