# Supplementary figures and images for: Genomic analysis of a novel pathogenic variant in the gene LMNA associated with cardiac laminopathies found in Ecuadorian siblings: A case report
Source: Front Cardiovasc Med. 2023 Mar 21;10:1141083. doi: 10.3389/fcvm.2023.1141083 (PMC10070725; doi:10.3389/fcvm.2023.1141083)

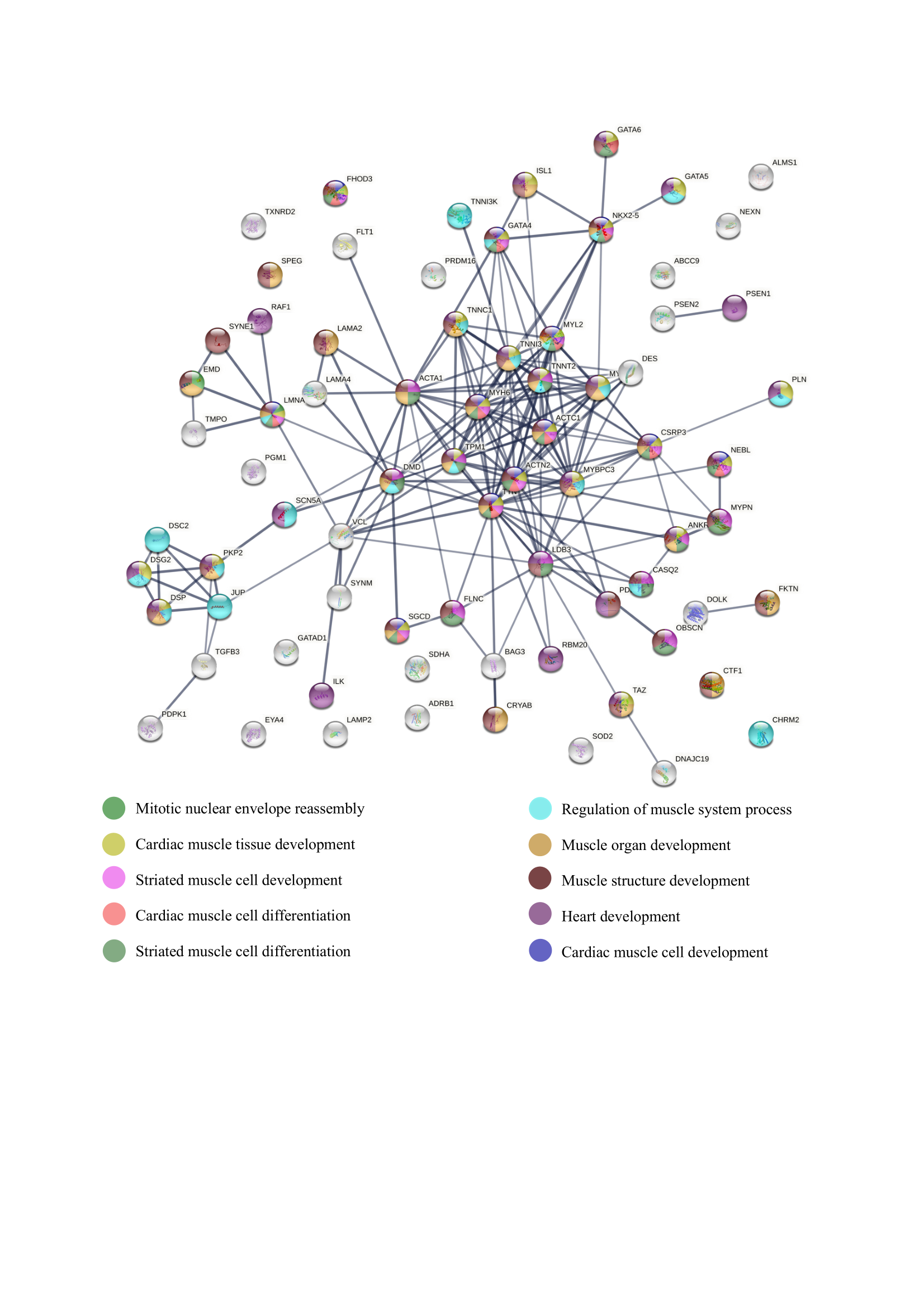

Supplement: Supplementary file 3 [file Image1.tiff]

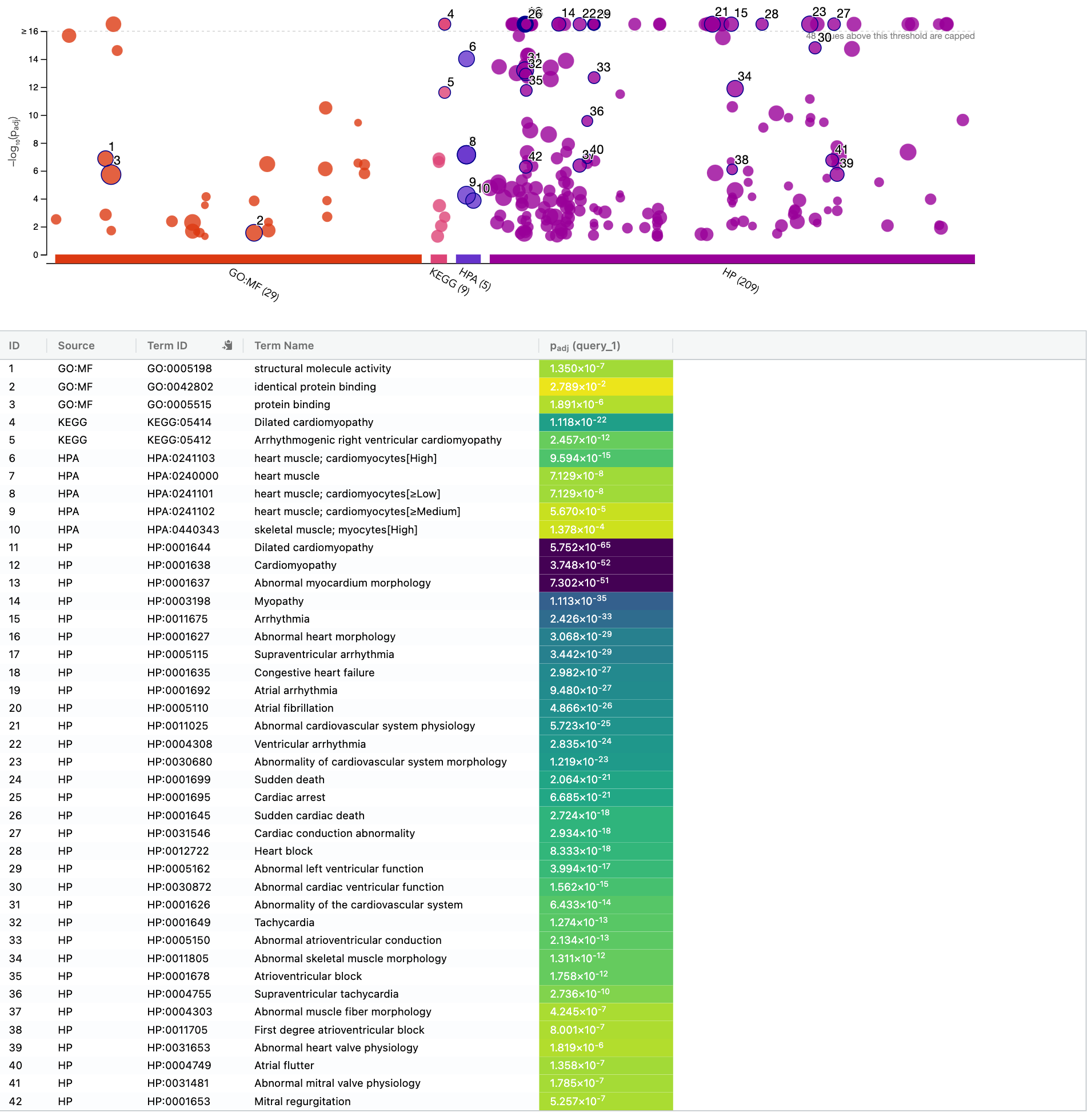

Supplement: Supplementary file 4 [file Image2.tiff]
